# A novel hypothesis for histone-to-protamine transition in *Bos taurus* spermatozoa

**DOI:** 10.1530/REP-16-0441

**Published:** 2016-12-21

**Authors:** Gerly Sillaste, Lauris Kaplinski, Riho Meier, Ülle Jaakma, Elo Eriste, Andres Salumets

**Affiliations:** 1 Competence Centre on Health TechnologiesTartu, Estonia; 2 Institute of Molecular and Cell BiologyChair of Bioinformatics; 3 Institute of Molecular and Cell BiologyChair of Developmental Biology, University of Tartu, Tartu, Estonia; 4 Institute of Veterinary Medicine and Animal SciencesEstonian University of Life Sciences, Tartu, Estonia; 5 Women’s ClinicInstitute of Clinical Medicine; 6 Institute of Bio- and Translational MedicineUniversity of Tartu, Tartu, Estonia; 7 Department of Obstetrics and GynecologyUniversity of Helsinki and Helsinki University Hospital, Helsinki, Finland

## Abstract

DNA compaction with protamines in sperm is essential for successful fertilization. However, a portion of sperm chromatin remains less tightly packed with histones, which genomic location and function remain unclear. We extracted and sequenced histone-associated DNA from sperm of nine ejaculates from three bulls. We found that the fraction of retained histones varied between samples, but the variance was similar between samples from the same and different individuals. The most conserved regions showed similar abundance across all samples, whereas in other regions, their presence correlated with the size of histone fraction. This may refer to gradual histone–protamine transition, where easily accessible genomic regions, followed by the less accessible regions are first substituted by protamines. Our results confirm those from previous studies that histones remain in repetitive genome elements, such as centromeres, and added new findings of histones in rRNA and SRP RNA gene clusters and indicated histone enrichment in some spermatogenesis-associated genes, but not in genes of early embryonic development. Our functional analysis revealed significant overrepresentation of cGMP-dependent protein kinase G (cGMP-PKG) pathway genes among histone-enriched genes. This pathway is known for its importance in pre-fertilization sperm events. In summary, a novel hypothesis for gradual histone-to-protamine transition in sperm maturation was proposed. We believe that histones may contribute structural information into early embryo by epigenetically modifying centromeric chromatin and other types of repetitive DNA. We also suggest that sperm histones are retained in genes needed for sperm development, maturation and fertilization, as these genes are transcriptionally active shortly prior to histone-to-protamine transition.

## Introduction

Evolutionary pressure has made spermatozoa motile streamlined cells whose nuclear material is compacted and protected during their journey to oocyte. This is mostly achieved by nuclear proteins, called protamines, which are specific only to mature spermatozoa. Protamines are synthesized during the elongating phase of spermiogenesis when extensive morphological, biochemical and physiological changes take place. Exchange of histones by protamines is a multistep process, which results in up to 20 times more compacted chromatin in sperms compared to somatic cells (Balhorn 2007). Chromatin compaction silences sperm gene expression until needed after fusion with oocyte, protects and maintains its DNA integrity in male and female reproductive tract and reduces the size of sperm head for better hydrodynamic properties (Braun 2001).

Although most sperm chromatin is packed with protamines, a portion of mature sperm DNA still remains associated with histones. An overall fraction of retained histones in mammalian spermatozoa has been shown to be between 1 and 15% (Gatewood *et al*. 1987, Hammoud *et al*. 2009, Erkek *et al*. 2013, Samans *et al*. 2014). Some of the histone variants found in murine and human sperm are also specific to testis and spermatozoa. These include both core histones such as TH2B, H2AL1, H2AL2, H3.3A and H3.3B (Govin *et al*. 2007) and linker histones H1T2 and HILS1 (Martianov *et al*. 2005) with various proposed functions. The current prevailing view is that histone-bound regions in spermatozoa are non-randomly distributed. Studies indicate, somewhat controversially, that nucleosomes are scattered across the genome but are enriched in certain regions. These include both gene-poor areas (Carone *et al*. 2014, Samans *et al*. 2014) and functional genomic regions such as promoters, transcription start sites and gene bodies (Arpanahi *et al*. 2009, Erkek *et al*. 2013, Castillo *et al*. 2014). Hammoud and coworkers (2009) demonstrated that in human sperm, differently modified histones remain at genes of embryonic development, like Homeobox gene cluster (Hammoud *et al*. 2009). However, the idea that histones are hallmarks for early embryonic gene activation was already proposed almost two decades ago (Gardiner-Garden *et al*. 1998) and could partly be supported by the idea that certain histones are transmitted from sperm to zygote (van der Heijden *et al*. 2008). Moreover, as an interesting finding, there seems to be a link between the amount of histones in sperm and transcriptional activity of early embryos (Ihara *et al*. 2014). At the same time, according to recent study, conserved histones rather occupy intergenic areas and repetitive elements (Samans *et al*. 2014). This coincides with immunostaining and hybridization studies that show nucleosomes in telomeres (Zalenskaya *et al*. 2000, Meyer-Ficca *et al*. 2013), subtelomeric and (peri)centromeric regions (Meyer-Ficca *et al*. 2013), and transposable elements such as long interspersed nuclear elements (LINEs) (Pittoggi *et al*. 1999). Nucleosomes are also believed to dominate in nuclease-sensitive areas between protamine toroids, by which DNA is attached to the nuclear matrix (Ward 2010). Given the controversial results in prevalence of histone-bound chromatin fraction in mammalian sperm cells and their possible localizations, further studies are needed to clarify this interesting phenomenon in mammalian reproduction.

By considering the aforementioned, our study had three goals. First, to find nucleosome conservation patterns in mature bull sperm cells in the samples from the same and different individuals. Second, to explore interindividual and intraindividual variance in nucleosome distribution in sperm samples. Finally, we aimed to propose a novel step-by-step substitution hypothesis for histone–protamine transition, which best describes our experimental findings.

## Materials and methods

### Biological material

The analyzed material was obtained from three Holstein bulls (Fag, Far and Ole) with controlled fertility. Three ejaculates were taken at different times from March to May from three animals (9 samples in total, Fag1, Fag2, Fag3, Far1, Far2, Far3, Ole1, Ole2 and Ole3).

All animal-related experiments are in agreement with EU directives (86/609/EEC).

### Chromatin fractionation and DNA extraction

The protocol for chromatin fractionation was adapted from Samans and coworkers (2014) with some modifications. First, to eliminate the effect of somatic cells, frozen semen samples were purified in BoviPure density gradient (Nidacon, Sweden), yielding ca 10–15 × 10^6^ sperm/mL. Cells were permeabilized with 0.5% Triton X–PBS–protease inhibitor cocktail (PIC) for 30 min on ice and centrifuged for 3 min at 10,000 RCF (4°C). The pellet containing nuclei was washed twice in PBS – 1× PIC solution, and next incubated with 10 mM dithiothreitol (Sigma Aldrich) to reduce intermolecular and intramolecular disulphide bonds. For nucleosomal isolation, nuclei were treated with 40 U of micrococcal nuclease (Thermo Scientific) during 2.5–3.0 minutes depending on enzyme activity at 37°C degrees and centrifuged at 10,000 RCF. As a result, two chromatin fractions were obtained: supernatant containing histones and pellet containing protamines. For DNA extraction, supernatant was incubated with proteinase K solution (200 µg/mL) for 3 h, followed by DNA extraction with phenol–chloroform and ethanol precipitation. DNA fragments were separated on 2% TBE (Tris/Borate/EDTA) agarose gel. Fragments corresponding to nucleosomal DNA (146 bp) were cut out of the gel and purified by using NucleoSpin Gel and PCR Clean-up kit (Macherey-Nagel).

### Analysis of nucleosomal fraction at the protein level

Histones and protamines from separated fractions were precipitated by using trichloroacetic acid. Next, material of 3 million sperm were treated in 2× tricine sample buffer at 95°C for 5 min and separated in 18% SDS-PAGE gel. For silver staining, the gels were treated with solutions from ProteoSilver Stain Kit (Sigma-Aldrich) according to manufacturer’s protocol. For Western blot, protein fragments were transferred to nitrocellulose (0.2 µm, Schleicher & Schuell) and polyvinylidene difluoride (0.2 µm, Bio-Rad) membranes by semi-dry transfer system. After blocking with 5% non-fat dry milk in TBS, membranes were incubated overnight at 4°C with primary antibody solutions 1:500 for histone H3 (Rabbit polyclonal antibody, ab18521, Abcam) and anti-protamine 1 (Mouse polyclonal antibody, ABIN519290, Abnova). After the washing step with TBST (Tween-TBS) membranes were incubated with secondary antibody 1:5000 solutions for histones (Goat anti-Rb IgG, horseradish peroxidase, HRP, ab97051 and Abcam) and protamines (Goat anti-mouse IgG, HRP, sc-2005, Santa Cruz) at RT for 2 h. Protein bands were amplified by using Amplified Opti-4CN Substrate Kit (170-8238, Bio-Rad) and detected using streptavidin-HRP-conjugated antibody 1:1000 and visualizing solutions according to manufacturer’s protocol.

### Library preparation for sequencing

Libraries were prepared using TruSeq ChIP Sample preparation kit (Illumina) according to the manufacturer’s instructions with the following modifications: reagents volumes were reduced by half, gel-based purification step was omitted and the PCR enrichment step consisted of 15 cycles. The quality of the libraries was assessed by analyzing on Agilent 2200 TapeStation instrument (Agilent). Samples were indexed during library preparation and pooled for multiplex sequencing. Sequencing was performed on the NextSeq 500 (Illumina) using NextSeq 500/550 High output v2 kit and producing 76 bp single-end reads.

### Bioinformatic analysis

All sequenced reads were mapped to *Bos taurus* reference genome (UMD_3.1.1/bosTau8, NCBI Accession GCF_000003055.5) separately for each sample, using bowtie2 (Langmead & Salzberg 2012) with default settings. The resulting SAM files were converted to BAM format using samtools (Li *et al*. 2009). From BAM files, the enrichment WIG files were generated with MACS 1.4 (Zhang *et al*. 2008). To conserve disk space and calculation time, only the coverage of each 10th genomic position (position 1, 11, 21 etc. of each chromosome) was recorded in enrichment file. Each row in WIG file gives a position in chromosome and a number of reads that were mapped to (overlapping) that position. As we expect that most reads were generated from purified nucleosomal DNA, we can interpret the number of reads for any given genomic position to correlate with the number of nucleosomes that were covering given position in purified sperm cells.

### GC content

GC content was calculated separately for all samples using raw sequencing reads.

### Creating subset of conserved positions

We created a subset of conserved histone-enriched positions in sperm cells by comparing all enrichment WIG files and extracting positions that had at least one read in all nine samples. For all matching positions, the average enrichment across all nine samples was calculated and recorded in composite WIG file. For subsequent analysis of histone content and distribution, this subset was used.

### Gene enrichment analysis

UMD_3.1.1/bosTau8 RefSeq gene annotation was downloaded from UCSC Genome Bioinformatics Site (http://hgdownload.soe.ucsc.edu/goldenPath/bostau/bigZips/). For each gene in database, we calculated the maximum and average enrichment values in the region starting from 1000 bp upstream of TSS (transcription start site) to 1000 bp downstream of TTS (transcription termination site). Functional profiling of the genes with highest maximum enrichment scores was done with g:Profiler tool (Reimand *et al*. 2007). To profile the genes whose paternal allele is active in early embryonic development for potential histone enrichment, we used the list of genes published by Graf and coworkers (2014).

### Calculating the histone conservation pattern relative to TSS

Using the gene annotation table, we calculated the average histone enrichment for each genomic position relative to TSS of all genes. This was done by iterating the relative distance from TSS from 5000 bp downstream to 5000 bp upstream, and for each relative distance value adding the enrichments of those genomic positions that were positioned exactly given distance from any TSS in annotation database. If given position was not recorded in composite WIG (i.e. the position 2–10), the enrichment value of the nearest recorded position was used. Then the averages were calculated by dividing the sums with the total number of TSS sites (genes).

### Calculating the histone conservation in repeats

UMD_3.1.1/bosTau8 RepeatMasker annotation was downloaded from UCSC Genome Bioinformatics Site (http://hgdownload.soe.ucsc.edu/goldenPath/bostau/bigZips/). For each repeat in annotation file, the average enrichment value in this region was calculated. Repeats were then grouped first by type, then by family and then by class and for each group the average, weighted by region lengths, were calculated.

### Comparison of histone conservation pattern in and between individuals

We calculated the correlation coefficients of the enrichment values for each genomic position in composite WIG file, between all pairs of samples.

### Analyzing the histone conservation pattern in genome

To analyze the histone conservation and substitution pattern for highly and moderately conserved positions, we first calculated the average enrichment of each genomic position in the three first samples from each individual (Fag1, Far1 and Ole1). These samples were prepared and sequenced separately and thus the potential batch effect between experiments was eliminated in both steps of analysis.

We then ordered all genomic positions by these average enrichment values (of the three samples) and separated deciles by cumulative enrichment. This means, the first decile contained all genomic positions that together made up 1/10 of total enrichment, the second decile contained the next 1/10 etc. Naturally the decile sizes were smaller for higher deciles. Next, we calculated the average enrichments in each decile using the enrichment values from the other six samples, by dividing the sum of all enrichment values with the decile size. We then calculated regression between the average enrichment in sample and average enrichment in decile, i.e. for each decile the actual enrichment was expected to conform to the following model:


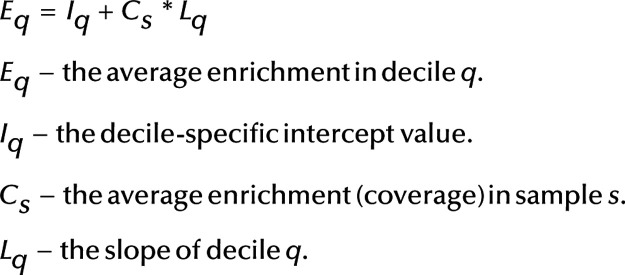



## Results

### Nucleosomal fraction contained histones

DNA extracted from histone fraction was separated on agarose gel, and the results were visualized. We noticed a clear band of 146-bp long DNA, which supposedly corresponded to mononucleosomal DNA (Supplementary Fig. 1A, see section on supplementary data given at the end of this article). In addition, a low-molecular DNA band (~50 bp) and high-molecular DNA band were detected. To confirm the proper fractionation of histone- and protamine-associated chromatin, we precipitated and separated proteins on polyacrylamide gel and performed silver staining and Western blot analysis. Silver staining showed low-molecular weight fragment corresponding to bovine protamine 1 (PRM1) 6.8 kDa (Consortium 2015) in protamine fraction, but not in nucleosome fraction (Supplementary Fig. 1B). We detected histone H3-specific band only in nucleosomal fraction and in semen lysate used as a control in Western blot analysis (Supplementary Fig. 1C). Contrary, PRM1-specific antibody gave a signal in protamine fraction and in control sample, but not in nucleosome fraction. Therefore, we concluded that nucleosomal fraction was enriched with histones.

### Histone coverage pattern indicated variability between samples

We analyzed altogether nine samples from three animals and three ejaculates. Sequencing reads were mapped to *Bos taurus* reference genome (UMD_3.1.1/bosTau8, NCBI Accession GCF_000003055.5) and the coverage for each 10th genomic position was calculated.

Overall, 162,504,196 recorded genomic positions, taken with 10 nucleotide interval, overlapped with at least one read in some sample. When normalized to all nucleotides, it corresponded to roughly 1.6 Gbp. 502,308 positions overlapped with at least one read in all samples, corresponding to roughly 5 Mbp. Although sparsely distributed single reads may be caused either by random preservation of single histones in sperm cells or by background noise of somatic cells, there were highly enriched regions, where the average number of reads was more than 100 times above the mean. These peaks probably corresponded to regions, where the majority of sperm cells in given sample retained histones.

The average sequencing coverage, normalized to full genome size, of samples varied from 0.043 to 0.406 (Table 1, row 1). We also calculated the average sequencing coverage of the top 5 percentile (by coverage) of all positions, separately for all samples (Table 1, row 3), ranging from 24.1 to 58.5. The maximum coverage for every sample is given in Table 1, row 2 (ranging from 71.0 to 136.0). As the results showed, the average sequencing depth (total amount of DNA) varied between samples remarkably more (9.4-fold) than the maximum and top 5th percentile (amount of DNA from the most enriched genomic positions; 1.9-fold and 2.4-fold, respectively). For the samples of the first ejaculate of each individual (Fag1, Far1 and Ole1), the higher average amount of sequenced DNA may be the result of batch effect in experimental procedure, as enzyme with lower activity and hence 30 s longer incubation time was used. For the remaining six samples that were purified and sequenced together, it may be either the result of experimental variance or due to the actual variance in histone content between the samples.

**Table 1:** The coverage averages, maximums and averages of top 5th percentile of all positions of all samples.^a^

	Sample								
	**Fag1**	**Fag2**	**Fag3**	**Far1**	**Far2**	**Far3**	**Ole1**	**Ole2**	**Ole3**
Average sequencing coverage	0.373	0.097	0.129	0.406	0.043	0.121	0.389	0.076	0.183
Maximum coverage	128	100	104	136	71	107	136	98	130
Average of top 5th percentile	46.8	45.1	46.4	51.9	24.1	30.2	58.5	37.5	45.8

a Sample names contain abbreviation of the name of the animal and sequence number of the ejaculate. Average sequencing coverage – the total length of mapped reads divided by the size of cow genome. Maximum coverage – the maximum number of mapped reads that overlap one genomic position. The top 5th percentile is calculated by cumulative coverage, i.e. those genomic positions with the highest coverage values that together sum up to 5% of total coverage.

**Figure 1:**
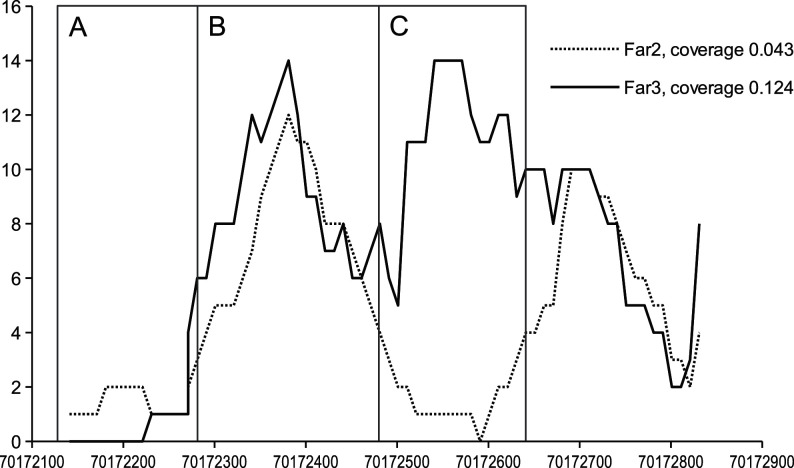
The variability of histone enrichment in specific positions. An example of genomic region (chromosome 11) showing the histone content in two different samples (Far2 and Far3). In certain regions, the enrichment is always low, in others always high (in both cases the correlation of enrichment with overall histone content of sample is low) and in some regions, the enrichment is correlated with the histone content of a given sample. *X*-axis – genomic position and *Y*-axis sample enrichment. A, B, C – different histone conservation patterns (A – always low, B – always high and C – correlated with average enrichment).

To find interindividual and intraindividual variance in histone pattern, we calculated the pairwise correlations between the coverage values of all samples for each 10th genomic position that had at least one read in each sample. The results are presented in Table 2. The correlation between coverages is higher for those samples that have more similar averages, regardless of whether these are from the same animal or different animals. Thus, we can infer that at least in given samples, the variance in histone pattern between different sperm samples from the same individual is not significantly lower than that between samples from different individuals. The lower correlation between samples with different average coverage indicated that the difference in coverage is not simply the result of smaller number of cells or smaller amount of purified DNA. There are probably certain differences in the histone conservation patterns between samples with smaller and higher amount of DNA. Therefore, it is possible that the samples with smaller overall amount of DNA (i.e. smaller coverage) had fewer retained histones in fewer places. An example of a genomic region with such pattern is shown in Fig. 1, region C for higher (Far3) and lower (Far2) amounts of DNA.

**Table 2:** The pairwise correlations of the coverages of genomic positions of all samples.^a^

	Fag1	Fag2	Fag3	Far1	Far2	Far3	Ole1	Ole2	Ole3
Fag1		0.669	0.663	0.892	0.695	0.790	0.878	0.664	0.817
Fag2			0.896	0.688	0.793	0.795	0.720	0.842	0.789
Fag3				0.695	0.786	0.798	0.726	0.844	0.797
Far1					0.757	0.810	0.900	0.698	0.828
Far2						0.826	0.755	0.807	0.804
Far3							0.793	0.785	0.856
Ole1								0.748	0.855
Ole2									0.821
Ole3									

a Only those positions where all samples had at least one read are included. The correlations are calculated between the enrichment values of all recorded genomic positions (number of reads overlapping this position) in two samples. Sample names contain abbreviation of the name of the animal and sequence number of the ejaculate.

### Histone retainment in functional regions of genome

The average GC content of sequenced reads was significantly higher than the genome average (51.8% and 41.8%, respectively). This indicates that histones are preferentially retained in GC-rich regions of the genome.

**Figure 2:**
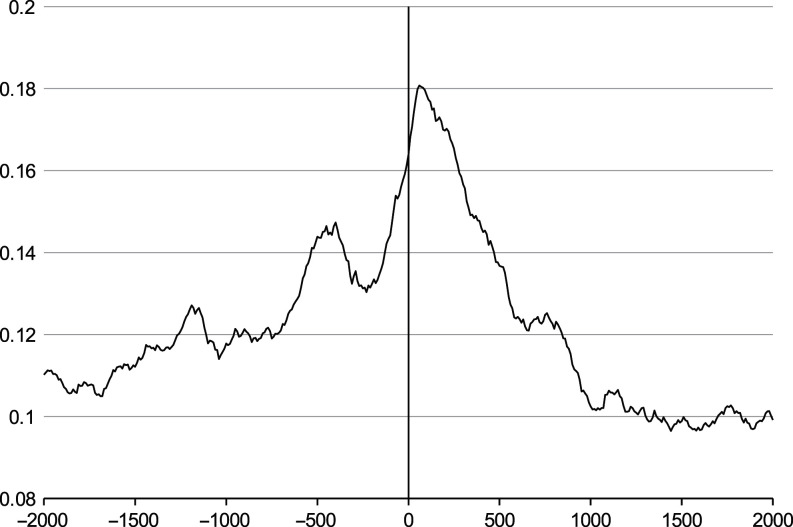
The average histone enrichment relative to transcription start site (TSS). The average histone enrichment relative to transcription start site (TSS). *X*-axis – the distance in base pairs (negative values represent upstream region) from TSS of known genes. *Y*-axis – the average enrichment by histones of all nine samples. Histone enrichment is the highest immediately after TSS. Another peak at −500 bp from TSS indicates on gene promoter or enhancer regions. Only positions from 2000 bp upstream to 2000 bp downstream are shown for clarity.

The average number of retained histones, relative to the position of TSS of annotated genes is shown on Fig. 2. There is clear enrichment peak immediately after TSS indicating that more histones are present in that region. Interestingly, there is another lower peak about 500 bp upstream from TSS, possibly indicating on the promoter or enhancer sequences.

### Histone enrichment on repetitive sequences

We found the average number of sequencing reads (histones) overlapping with known repeating element in bovine genome, grouped by class, family and type. Of all repeat classes, satellite DNA, represented by a family of centromeric repeats, had the highest enrichment score (4.9-fold) (Table 3; Supplementary Table 1, with family information). Next highly represented classes included rRNA and SRP RNA repeats (Table 3). Also ERVK (endogenous retrovirus group K), a group of transposable elements containing LTR-s (long terminal repeats), was enriched in our dataset (Supplementary Table 1). All these enrichments were robust, i.e., they were detectable in both full dataset and each individual sample, regardless of overall coverage value in that sample.

**Table 3:** Repetitive sequence classes categorized by histone enrichment score.^a^

Class	Enrichment score	Number of copies	Overall length in bp
Satellite	4.9288	9481	10,216,800
rRNA	0.9225	1432	267,280
SRP RNA	0.5476	68	13,150
SINE	0.1635	2,157,060	341,045,070
RNA	0.1404	337	54,870
LTR	0.1375	538,411	134,180,980
tRNA	0.1299	2632	194,700
LINE	0.0992	1,888,497	772,366,750
scRNA	0.0954	31	2760

a The average enrichment is calculated by dividing the total number of histones overlapping given repeat types with the total length of these repeats in genome.

LINE, Long Interspersed Elements; LTR, Long Terminal Repeats; rRNA, ribosomal RNA; scRNA, small cytoplasmatic RNA; SINE, Short Interspersed Elements; SRP RNA, Signal recognition particle RNA; tRNA, transfer RNA.

### Histone-enriched genes

We calculated the average and highest histone enrichment for all annotated genes in bovine genome, including 1000 bp upstream from TSS and 1000 bp downstream from TTS (Supplementary Table 2). We performed functional profiling with g:Profiler (Reimand *et al*. 2007) with the lists of top 100 and top 200 genes with the highest maximum enrichment values. The only pathway that was significantly overrepresented in both lists was cGMP–PKG signaling pathway. Corresponding *P* values were 9.60E-04 for the list of 100 genes and 2.87E-06 for the list of 200 genes. Neither generic spermatogenesis term (GO:0007283) nor any of its 10 sub-functions in Gene Ontology database had significant enrichment in our lists.

We also tested whether any known imprinted genes have above-average histone enrichment. For that we used bovine imprinted gene list from Geneimprint (http://www.geneimprint.com) and looked up the maximum and average read count for each of those genes from the full gene enrichment table, but resulting in no detectable enrichment.

### The relation of histone conservation and embryogenesis

Graf and coworkers (2014) identified in their study 937 genes transcribed from the paternal allele during different time points of bovine embryogenesis. Of these, we found histone retainment in 21 genes. Relying on expression data of the same publication, nine of these histone-associated genes are first expressed in 8-cell embryo, five in 16-cell embryo and seven in blastocyst. The results are depicted on Supplementary Table 3. Based on these results, we cannot conclude that sperm-derived histone-associated genes are needed for early embryogenesis.

### Histone conservation pattern

**Figure 3:**
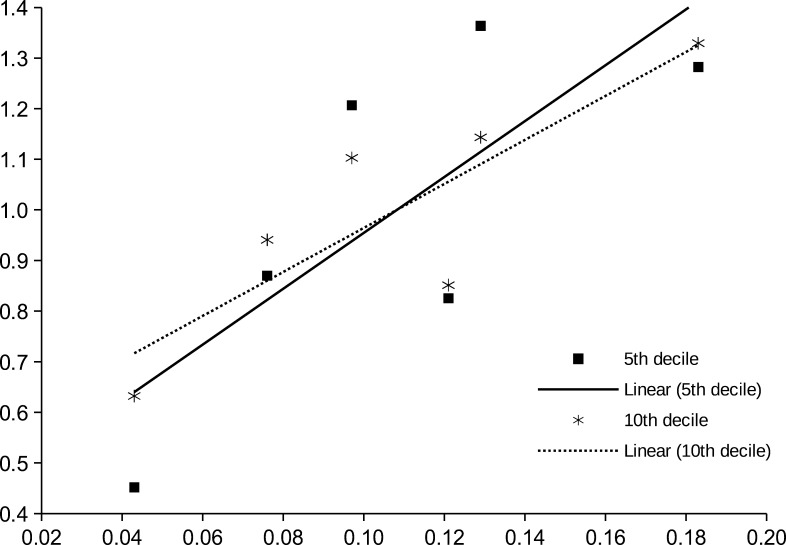
The correlation of histone enrichment in specific positions with the average histone content of samples. Dots – individual samples. Lines – linear regression lines. Only 5th and 10th deciles by cumulative enrichment are shown. Deciles are calculated by different samples than regression values. The higher slope of the regression line of the 5th decile indicates that in these regions the enrichment is more strongly correlated with the histone content. *X*-axis – histone content (sequencing coverage) of sample. *Y*-axis – the average normalized enrichment in given decile.

To understand the histone enrichment pattern, the dataset was split into deciles based on the average enrichment in control group (Fag1, Far1 and Ole1). For each decile, the average in test group (Fag2, Fag3, Far2, Far3, Ole2 and Ole3), regression and correlation coefficients between the average enrichment in a given decile and test group average enrichment were calculated. The results are presented in Table 4. The regression coefficients (intercept and slope) are normalized by test group average, to highlight the variance. As we can see, the normalized regression slope is the highest for the 5th decile, descending for both lower and higher deciles. This means that for genomic positions in given decile, the relative number of retained histones is most strongly influenced by the overall genomic average (sequencing coverage). To illustrate this feature, the slopes of 5th and 10th percentiles are shown on Fig. 3.

**Table 4:** Averages, regression and correlation values of deciles by coverage.^a^

Decile	Fag1	Fag2	Fag3	Far1	Far2	Far3	Ole1	Ole2	Ole3	Test Avg	Positions	Intercept	Slope	Correlation
1	3.79	5.43	5.98	4.7	2.13	3.42	5.29	3.95	4.99	4.32	62095	0.535	4.299	0.626
2	6.85	8.05	9.08	8.46	3	5.21	9.46	5.75	7.9	6.5	34542	0.459	4.999	0.693
3	8.92	9.76	11.1	10.98	3.59	6.4	12.32	6.97	9.93	7.96	26558	0.427	5.299	0.723
4	11.04	11.51	12.96	13.63	4.23	7.63	15.26	8.19	11.88	9.4	21430	0.416	5.401	0.74
5	13.67	13.25	14.97	16.73	4.96	9.06	18.53	9.55	14.08	10.98	17490	0.402	5.526	0.766
6	17.17	15.69	17.56	20.51	6.05	11	22.74	11.39	16.86	13.09	14165	0.407	5.484	0.782
7	21.84	18.69	20.81	25.73	7.88	13.5	28.51	14.17	20.89	15.99	11247	0.422	5.346	0.808
8	28.45	23.57	25.28	32.9	11.41	17.33	36.56	18.9	27.09	20.6	8739	0.474	4.86	0.825
9	35.63	31.74	33.07	41.82	17.05	22.27	46.98	26.34	35.3	27.63	6877	0.56	4.066	0.769
10	51.17	40.98	42.49	57.96	23.49	31.63	65.04	34.96	49.4	37.16	4914	0.53	4.348	0.854

a Deciles are calculated by the average coverage value in the control group (Fag1, Far1 and Ole1). Test Avg – the average coverage in test group (Fag2, Fag3, Far2, Far3, Ole2 and Ole3). Positions – the number of positions in given decile. Intercept and Slope – regression values, divided by Test Avg. Correlation – the correlation coefficient between the average coverage of full genome and Test Avg. Sample names contain abbreviation of the name of the animal and sequence number of the ejaculate.

## Discussion

The presence of histones in mature mammalian spermatozoa has been known for decades. Yet, their amount, distribution and possible function are still a matter of debate. As protamines are removed after fertilization (Nonchev & Tsanev 1990), they are apparently needed for secure transport of genetic material. This leaves a question, among others, whether retained histones have any specific roles or are they randomly retained after protein transition in spermiogenesis? One of our goals was to describe histone conservation by exploring histone distribution in and between individual animals. To our knowledge, such data have not been published, although nucleosome preservation between species has been recently reported (Samans *et al*. 2014).

We analyzed three separate ejaculates from three fertile bulls and noticed nine-fold difference in sequencing coverage. It implies that there was similar variance in the number of nucleosomes that were extracted and purified from samples. This variance could be explained either by the different amount of preserved histones in samples or alternately by the variance in sample preparation (e.g. incomplete fractionation or different MNase activity or incubation time).

By comparing the highest coverage 5th percentile of genomic positions of all samples, we found that the variance was still present, although significantly lower (about 2-fold). We expect that if experimental variance would influence all genomic regions by a similar fraction, the relative difference in enrichment should remain similar in all percentiles. Also, if the variance was caused by different MNase activity, as recently shown by Mieczkowski and coworkers, it could not explain the relative uniformity in highest percentiles (Mieczkowski *et al*. 2016). Taking into consideration the higher variance in average sequencing coverage (9.4-fold) rather than in maximum (1.9-fold) or average of top 5th percentile coverage (2.4-fold), the variance of sequencing coverage across the samples cannot be explained thoroughly by experimental procedure and more likely reflects the real biological differences.

Another potential source of the variance in histone content could be background noise from the histones of somatic cells. This could mean that the regions with the highest coverage indicate the relative number of sperm cells in samples (2-fold variance between the samples), while the overall histone content represents the amount of somatic cell contamination. Our analysis of correlation between the number of sequencing reads in all genomic positions between all samples indicated that correlation was higher if the coverages were more similar and lower if they were different. This finding rules out the overall somatic contamination, as somatic background should be distributed more randomly. Thus, the correlations between the samples with high histone content (i.e. with more contamination) should be lower. The explanation of the observed correlation pattern instead suggests that highly covered samples contain more peaks, not simply higher peaks. Further confirmation of the difference in the histone conservation pattern between samples is how the histone content of different coverage deciles depends on the overall histone content. The regions that correspond to most enriched areas (upper deciles) retain their histones in most samples. The regions in the middle deciles, i.e., moderately enriched areas, retain histones only if the overall histone content is high and lose these if it is low. One such example region with such histone conservation pattern, for two samples, is presented in Fig. 1. The areas A and B have similar amount of conserved histones in both samples (high and low coverage), whereas the region C has significant number of conserved histones only in the sample with higher coverage. Therefore, we suggest that, based on our data, the different genome regions have different affinity toward the histone complexes and gradual decrease of histone content from different regions can be suspected. This may be caused by step-by-step procedure of histone–protamine transition, where the easily accessible regions are substituted first (area A on Fig. 1). The substitution then proceeds by replacing histones in less accessible regions (area C), whereas certain regions always retain histones (area B). This process may remain incomplete for unknown biological reasons, resulting in sperm cells that have higher average histone content. Thus, the average content of histones can vary significantly between sperm samples even from the same individual, explaining the wide differences in histone content reported by literature. If this hypothesis will be confirmed, it may open interesting areas of study relating the biological factors during spermatogenesis to histone retainment and potentially to the fertilization potential of sperm. However, which are the factors that influence the histone content and retainment in mature sperm cells and lead to interindividual and intraindividual differences between the semen samples are unclear.

Our analysis of repetitive sequences showed significant histone enrichment in the satellite DNA of centromeres. This is in agreement with previous findings (Samans *et al*. 2014) and supports the notion that centromeric sequences contribute into nucleosome stability in mouse metaphase chromosomes (Widlund 1998). One of the centromere-binding proteins, CENP-A (centromere-specific protein A), is present in mature mammalian spermatozoa and has been proposed to organize the structure of paternal genome in early embryogenesis (Palmer *et al*. 1990). In sperms, centromeric sequences located in equatorial segment under the region of plasma membrane, which first fuses with oocyte (Powell *et al*. 1990). Histone modifications in sperm heterochromatin possibly carry information about chromatin structure pattern into early embryo as these marks are removed after pronucleus formation (van der Heijden *et al*. 2006).

Another group of repetitive elements which in our study turned out to be associated with histones were genes of ribosomal RNA, also known as ribosomal DNA. To our knowledge, this finding has not been previously reported. Genes for rRNA are arranged in tandem repeat clusters found in telomeric ends (Nadel *et al*. 1995*b*) and spots coinciding with centromeres (Powell *et al*. 1990). Also, in hamster sperm, 5S rRNA clusters seem to be organized into small DNA loop domains that attach to nuclear matrix (Nadel *et al*. 1995*a*). These domains, also known as nuclear matrix attachment regions, have a structural role for chromatin compaction as well as they are foci of replication and transcription in zygote. Because of sensitivity to nuclease digestion, these regions are likely bound by histones (Ward 2010). In the light of this knowledge, our results of rRNA genes being nucleosome bound seem to be possible. It is also interesting that DNA sequences of sperm rRNAs are assumingly among the first ones to be decondensed and transferred into oocyte (Powell *et al*. 1990). In addition, transcription from rRNA genes is detected already in 4-cell bovine embryo (Viuff *et al*. 1998). Thus, relying on previous studies, we speculate that histones may mark for DNA loops, which contain genes of rRNA-s. As these domains attach to nuclear matrix that provides proper formation of male pronucleus and DNA replication (Shaman *et al*. 2007), sperm histones may rather play structural role from the aspect of early embryogenesis. Interestingly, our results also show that genes of RNA component of signal recognition particle (SRP) are histone-enriched in sperm. SRP is a ribonucleoprotein complex that facilitates sorting and translocation of membrane and secretory proteins (Jacobson & Pederson 1998). To our knowledge, this is the first report to describe connection between histones and genes of SRP RNAs in eukaryotes.

However, some caution is needed while interpreting these results because repeat regions are known to cause problems for both genome assembly and mapping. First, there may be not enough sequence difference to unambiguously locate certain read on genome. Also, the copy numbers of tandem repeats may vary because the deletions and duplications of repeated segments are more common compared to other genomic regions. This can influence the estimated amount of conserved histones in repeat regions. The above-average number of reads mapped to certain segment may be caused by mapping bias where one repeated segment out of many similar ones is preferred by heuristic algorithm. Or alternately there may be variance between the number of copies of this segment between individuals, so that the individual with higher repeat copy number shows in analysis as having larger amount of conserved histones. The effect of mismapping was eliminated in our study by calculating only the average histone enrichment of repeats, by taking all annotated locations of these repeats into account. The possible inter-individual variance remains still one possible cause of overestimation or underestimation of the histone enrichment of repeated regions.

Some studies have indicated that histones in sperm are retained in the genes of embryonic development (Gardiner-Garden *et al*. 1998, Hammoud *et al*. 2009). Paternal genome undergoes major changes after fertilization. Prior to first replication, protamines are replaced by maternal histones (Nonchev & Tsanev 1990) and paternal genome is demethylated, leaving regions such as imprinted genes, centromeres and retrotransposons unaffected. Overall methylation pattern is re-established in bovine 16-cell embryo (Dean *et al*. 2001), and this coincides with the major genome activation at the stage of 8–16 cells (Frei *et al*. 1989). Yet, some transcriptional activity is described already in 1-cell zygote and 2-cell bovine embryos (Memili & First 2000). Until the embryo genome activation, the development is dependent on maternal transcripts. Therefore, if the conserved sperm histones have a functional role in oocyte, this would be needed at very early stage of embryogenesis. Indeed, as already discussed, histones may play role in zygotic chromatin structure (van der Heijden *et al*. 2006, 2008), but it is not evident whether zygotic transcription itself is affected by the content of sperm histones at this time as its genome is still mostly quiescent. It is although conceivable that paternal histone modifications are retained during cell replication and enable (or hinder) transcription after embryonic genome activation as described in Paradowska and coworkers (2012). This would explain our finding of close downstream region of TSS being clearly enriched in sequenced DNA. Also, a higher peak was found in 500 bp upstream from TSS, possibly in promoter or enhancer areas. From our comparison of sequencing coverage with the list of known bovine paternal transcripts, we found only 21 out of 937 genes to have significant enrichment and none of them are expressed earlier than in 8-cell embryos (Graf *et al*. 2014). Based on this result, we cannot conclude that histone-bound genes are specifically needed for early embryogenesis.

We calculated the average and maximum sequencing coverage for functional regions of all annotated genes (1000 bp from TSS, including gene body and 1000 bp from TTS). The maximum coverage (maximum peak) could be interpreted as the most enriched region within the area of interest. The higher the maximum peak, the more histones were found from all nine samples in one specific position. High average coverage would rather imply semi-uniform distribution of histones within the region, with no indication of more conserved area within gene. We compiled top 100 and top 200 gene lists from these data. Functional analysis revealed cGMP–PKG pathway (KEGG:04022) (Kanehisa *et al*. 2016) to be statistically significant among the list of top 100 (9.60E-04) and top 200 genes (2.87E-06). Protein kinase G (PKG) is a Ser/Thr-specific kinase that phosphorylates its target molecules upon activation by cGMP. Although being a common signaling pathway in cells, it appears that in human sperm, cGMP–PKG mediates progesterone-induced chemotaxis to oocyte (Teves *et al*. 2009), sperm motility (Miraglia *et al*. 2011) and acrosome reaction in response to nitric oxide (Revelli *et al*. 2001) and is involved in calcium influx regulation during capacitation in mice (Cisneros-Mejorado *et al*. 2014).

Among cGMP–PKG pathway genes with higher values of histone maximum peak, we found signal transducers such as PLCB1 (phospholipase C beta 1) needed for calcium release in acrosome reaction (Walensky & Snyder 1995) and genes encoding subunits of different ion transporters. To mention a few, ATP1A1 (ATPase Na+/K+ transporting subunit alpha 1) and ATP1A4 (ATPase Na+/K+ transporting subunit alpha 4) described in signal cascade during bovine sperm capacitation (Newton *et al*. 2010). Also, ATP2B4 (ATPase plasma membrane Ca^2+^ transporting 4) to provide sperm hyperactivated motility for fertilization (Okunade *et al*. 2004) and ATP2B1 (ATPase plasma membrane Ca^2+^ transporting 1) whose knockout leads to embryonic lethality (Prasad *et al*. 2004).

Also, although spermatogenesis’s (GO:0007283) biological process was not statistically significantly overrepresented in our dataset, there nevertheless were several interesting genes that appeared to be histone bound in our top 100 and top 200 gene lists. We found the highest maximum peak value (104.2) for SUFU (supressor of fused homolog), a negative regulator of Hedgehog (Hh) signaling pathway. Hh mediates normal development in embryonic and adult tissues and is found in both vertebrates and invertebrates. For example, in mammals, it plays a role in male germline differentiation (Bitgood *et al*. 1996) and several stages of spermatogenesis (Morales *et al*. 2009). It appears that SUFU becomes detectable in elongating spermatids in murines and possibly switches off Hh signaling pathway (Makela *et al*. 2011). Therefore, it would be reasonable that SUFU remains histone associated as it was transcribed in final stages of spermatogenesis. Another interesting finding with the second highest maximum peak value (92.7) and significantly high histone coverage (4.95) was RNF122 (RING finger protein 122). RNF proteins are mainly known as ubiquitin ligases. Although little is known about RNF122 (Peng *et al*. 2010), some members of RNF proteins are shown to be present in murine-elongating spermatids where they possibly participate in processing of misfolded proteins (Nian *et al*. 2008), acrosome biogenesis, head development and formation of tail–head coupling apparatus (Rivkin *et al*. 2009). Among genes with high average histone coverage, TSPY (testis-specific protein, Y-linked) was significantly enriched (9.2). In humans, TSPY locates in tandem repeats, adjacent to centromeric region of short arm of Y chromosome. TSPY is expressed in fetal prespermatogonia (Honecker *et al*. 2004) as well as in spermatogonia and spermatocytes of adult testis (Schnieders *et al*. 1996, Lau *et al*. 2011). It binds to cell cycle controlling cyclins, and therefore contributes into cell proliferation and renewal (Li & Lau 2008, Lau *et al*. 2011). In humans, the low copy number of TSPY repeats is in correlation with poor sperm production and male infertility (Giachini *et al*. 2009).

To summarize, based on the analysis of nine sperm samples, our study indicates that there are regions that preferentially remain histone bound. Although there is variance in histone conservation pattern, which we speculate is derived from patterns of histone–protamine exchange, the interindividual and intraindividual differences are similar. Our enrichment analysis showed that sperm histones are retained in repetitive elements – centromeres, genes of rRNAs and SRP RNAs. To our knowledge, last two elements are a new finding in the context of sperm histone retainment. Also, our study revealed that genes of cGMP–PKG pathway were overrepresented in our dataset. As this pathway is also involved in signaling during sperm chemotaxis, capacitation and acrosome reaction, we propose that histones are retained in genomic areas needed for successful sperm maturation and fertilization. This is also supported by our finding of several spermatogenesis-associated genes to be histone enriched. To the contrary, we did not find any correlation between histone content of sperm and early embryonic gene expression. Therefore, we speculate that histones may have a structural role, as associated with repeated genomic regions, like centrosomal chromatin, and they may contribute epigenetic structural information into early embryo. As another aspect, we suggest that sperm histones are retained in genes needed for sperm development, maturation and fertilization as these genes are transcriptionally active shortly before histone-to-protamine transition.

## Supplementary data

This is linked to the online version of the paper at http://dx.doi.org/10.1530/REP-16-0441.

## Declaration of interest

The authors declare that there is no conflict of interest that could be perceived as prejudicing the impartiality of the research reported.

## Funding

This study was supported by the Estonian Ministry of Education and Research (IUT34-16 and IUT8-1), Enterprise Estonia (EU48695), grants from European Union’s FP7 Marie Curie Industry-Academia Partnerships and Pathways (IAPP, SARM, EU324509) and Horizon 2020 innovation programme (WIDENLIFE, 692065).
